# Association between Compliance with the 24-Hour Movement Guidelines and Fundamental Movement Skills in Preschoolers: A Network Perspective

**DOI:** 10.3390/ijerph17155443

**Published:** 2020-07-28

**Authors:** Clarice Maria de Lucena Martins, Cain Craig Truman Clark, Paulo Felipe Ribeiro Bandeira, Jorge Mota, Michael Joseph Duncan

**Affiliations:** 1 Department of Physical Education, Federal University of Paraiba, João Pessoa-PB 58000-000, Brazil; 2 School of Health Life Sciences, Coventry University, Priory Street, Coventry CV1 5FB, UK; ad0183@coventry.ac.uk; 3 Department of Physical Education, Universidade Regional do Cariri – URCA, Crato 63105-000, Brazil; paulo.bandeira@urca.br; 4 Centre of Physical Activity, Health and Leisure, Faculty of Sport Sciences, University of Porto, 4500 Porto, Portugal; jmota@fade.up.pt; 5 Centre for Applied Biological and Exercise Sciences, Priory Street, Coventry CV1 5FB, UK; aa8396@coventry.ac.uk

**Keywords:** 24-h movement behaviors, fundamental movement skills, network perspective

## Abstract

The present study aimed to analyze the compliance with the 24-h movement guidelines and its association with fundamental motor skills (FMS) in early childhood, considering sex and Body Mass Index (BMI) in a network perspective. Two hundred and twelve preschoolers (109 boys, 4.45 ± 0.78 years old) provided physical activity (PA), sleep duration, screen time, fundamental motor skills (FMS) and BMI data. Relationships between compliance with movement behaviors guidelines, FMS, sex and BMI were calculated using a network analysis. Only two percent of the entire sample complied with the overall 24-h movement behaviors recommendations; while the emerged network in the present study emphasized ball skills as the most critical centrality variable, according to age, reinforcing the importance of ball skills for the engagement and maintenance of PA in children. The present study presents a novel statistical and theoretical perspective that permits hitherto unseen insight into the associations between movement behavior, FMS and their correlates, that appropriately consider the inherent, multifaceted, complexity of these relationships.

## 1. Introduction

The impact of inadequate time spent engaged in physical activity (PA), sedentary behaviors (SB) and sleep resulting in negative health outcomes is well established [1]. In children under five years-old, physical inactivity is associated with obesity indicators, poor skeletal, cardiometabolic and cognitive and motor skills profiles [2]. Conversely, the benefits of these behaviors in young children are evidenced across empirical investigations [1] and are key tenets of short and long-term health outcomes [3].

The most recent World Health Organization (WHO) 24 h guidelines on Movement behaviors for the Early Years [3] recommend that, for preschoolers, a healthy 24 h during the day includes: (i) ≥180 min of PA, including at least 60 min of energetic play, (ii) ≤1 h of sedentary screen time and (iii) between 10 and 13 h of good quality sleep. Nonetheless, besides the evident benefits of a healthy lifestyle, a large proportion of young children worldwide do not engage sufficiently in moderate-to-vigorous physical activity (MVPA) have excessive exposure to SB and do not accrue adequate amounts of sleep [4,5,6,7]. This is particularly important, considering that behaviors established in childhood have repercussions throughout life.

Theoretical models suggest that PA and fundamental movement skills (FMS), operationally defined as the basis of more complex movements required to participate in sports, games or other context specific PA [8], are reciprocal and dynamically related [9,10]. Stodden and his colleagues [10] postulate that young children’s PA may drive their development of FMS. Indeed, current available evidence shows that low levels of adherence to PA guidelines in childhood may be related to low levels of FMS, although the degree of evidence is weak to moderate [11,12]. Moreover, results from a recent systematic review reported no significant association between light physical activity (LPA) and FMS and an uncertain evidence for the relationship between locomotor skills and MVPA [13]. There is also evidence that preschoolers who spend excessive time in SB are more likely to have lower FMS scores [14]. In addition, it is well known that achieving an adequate amount of sleep plays a fundamental role in memory consolidation process and acquisition and retention of information [15]; where inadequate sleep may negatively impact on FMS acquisition. Indeed, a delay in the development of FMS has been postulated in children that do not accrue adequate time engaged in PA and sleep and spend excessive time sedentary. Thus, understanding the relationship between the adherence to the milieu of movement behaviors (PA, sleep, SB) and FMS may yield insights into future health. However, while such information would be invaluable to teachers, physical educationalists, health professionals and researchers, such relationships remain relatively unexplored; where the complexity and nonlinearity of the interrelated variables contributes to the dearth of available literature.

A recent conceptual model recognizes that, besides the potential cultural and geographic specificity of motor skills, personal attributes, such as weight status, can impact on motor skill development through the lifespan [9] and should be incorporated as a key tenet to consider within any model used to understand FMS trajectories. Thus, the relationship between these variables may be better understood as a network, interacting to form an emerging pattern that allows the identification of those variables that are most important to maintain a desirable theoretical pattern of the system [16,17]. The network perspective allows us to assess the role of each variable within the system from the measures of centrality, indicating which variables have the strongest associations, and those that are more sensitive to changes from interventions. Indeed, no study to date has considered such a network analysis approach in the context of movement behaviors and FMS in early childhood, and this study aims to analyze the compliance with 24-h movement guidelines and its association with FMS in early childhood, considering sex and BMI.

## 2. Materials and Methods

### 2.1. Study Sample

For this cross-sectional study, preschool children aged 3–5 years, of both sexes, and registered in early childhood education and care services (ECEC) of João Pessoa, a large seaside city in the northeast of Brazil, were eligible.

The preschool public education zone is organized in nine districts, where eighty-six ECEC are located. From those, fifty institutions located in six districts have children aged 3–5 years registered as students. For the purpose of this study, we randomly selected one ECEC from each educational district, which corresponds to 573 preschoolers of different ages. A total of 276 eligible children were invited to participate, of which, 64 (23%) did not provide consent, did not provide valid accelerometer data, did not have sleep/screen time information or did not participate in TGMD-2 assessments. Therefore, the final sample included 212 preschool children with complete movement behaviors and motor skills data.

### 2.2. Data Collection

#### 2.2.1. Procedures

All the schools’ staff of the six preschools and parents were informed about the research’s goals, protocols and procedures in meetings with the project coordinator (one session in each school) and agreed to participate in the present study. Trained physical education teachers and graduate students conducted the assessments.

The school administration provided all sociodemographic data (children’s age, birth date, parent’s contact and address). Parents were invited for a meeting at school and were interviewed individually. Screen and sleep time were collected during this interview. Parents were also informed about the accelerometer use.

Assessments were conducted during a four-month period. Anthropometric data and FMS were assessed at preschools, and the accelerometer was placed on the participating children, who used it over 7 days.

#### 2.2.2. Measurements

a) Anthropometric Measures:

Height (cm) and body mass (kg) were assessed using a *Holtain* stadiometer and weigh scale (Seca 708, Germany), while the participant was lightly dressed and barefoot. BMI was calculated by dividing body weight with the squared height in meters (kg/m^2^) [18].

b) Physical Activity:

PA was objectively measured using accelerometers (Actigraph, model WGT3-X, Pensacola, FL, USA), a valid instrument for measuring PA in preschoolers [19]. The preschool teachers of the EECC’s received training (verbal and written instructions) for the correct use of the accelerometer, including placement. The participants were instructed to wear the accelerometer on the right hip for seven consecutive days (Wednesday morning to Tuesday afternoon). Children were allowed to remove the device during water-based activities and while sleeping (at night). During the preschool time, accelerometers were removed by teachers around 11 am for children’s bath and attached properly after it. Parents were also instructed to remove the belt during night and attach when children woke up.

The device initialization, data reduction and analysis were performed using the ActiLife software (Version 6.13.3) (Pensacola, FL, USA). Accelerometers were set up to measure ActiGraph counts considering vector magnitude and using a 15 s epoch length [20]. Periods of ≥20 min of consecutive zero counts were defined as non-wear time and removed from the analysis, and the first day of accelerometer data were omitted from analysis to avoid subject reactivity [21]. Valid data were considered for a minimum of 8 h of wear time, during three days (one weekend and two week days). The total average wear time was 10.9 h/day (SD ± 1.4 h of wear time between children).

Time spent in the commonly defined intensity domains; light, moderate and vigorous was estimated using the cut-points proposed by Butte et al. [22], for vector magnitude, with light–intensity defined as 820–3.907 counts, moderate-intensity defined as 3908–6.111 counts and vigorous-intensity as ≥6.112 counts. The amount of time spent sedentary was estimated using the 819 counts/15 s cut-point.

c) Sleep Time:

Parents reported children’s usual daily sleet hours and screen time. This approach has been validated against estimates from sleep logs of objective actigraphy in young children [23]. Parents were asked to recall the total average hours their child slept as follows: “On weekdays, how many hours of sleep does your child usually have during the night?” and “On weekend days, how many hours of sleep does your child usually have during the night?”. The questions were separate for weekdays and weekend days and reunited to analysis. Overall, sleep hours were calculated as follows: ((Sleep on weekdays × 5) + (Sleep on weekend days × 2))/7. Finally, the results were multiplied by 60 to represent minutes per day.

d) Screen Time:

Parents were also asked to recall the total average duration their child watched TV, used the computer and used videogames. The questions addressed weekdays and weekend days separately and were combined for analysis (Cronbach’s α = 0.87). For screen time the questions were: “How many hours during a week day does your child usually watch TV, use computer, smartphones or electronics games?” and “How many hours during a weekend day does your child usually watch TV, use computer, smartphones or electronics game?”. Then, the same procedure used for sleep hours was applied.

e) Fundamental Movement Skills:

Fundamental movement skills were measured using the Test of Gross Motor Development—Second Edition (TGMD-2) [24]. The TGMD-2 is valid and reliable for use in Brazilian children [25]. This test evaluates gross motor performance in children aged 3–10 years [25] and consists of two factors: six locomotor skills (run, gallop, hop, leap, jump and slide) and six ball skills (strike, bounce, catch, kick, throw and underhand roll).

The TGMD-2 was administered at each preschool, according to the recommended guidelines [24]. Before the testing of each skill, participants were given a visual demonstration of the skill by the researcher using the correct technique but were not told what components of the skill were being assessed. Participants were then called individually to the practice trial. After that, participants performed the skill twice. General encouragement, but no verbal feedback on performance was given during or after the tests. All skills were video-recorded and later assessed by one trained assessor who have not administered the tests. The time taken to assess each child was approximately 40 min.

Using the media player classic software, a total of 4896 videos were analyzed to evaluate skills’ criteria. Two Professors in the motor behavior field, with experience in assessing the TGMD-2, carried out a training process on the protocol’s criteria with a master student who did not participate in data assessment. The training process was carried out over two weeks and 10% of the videos were randomly analyzed twice by the evaluator, with an interval of ten days between each evaluation, to determine the intraclass correlation coefficient (ICC). A high agreement for the locomotion score: ICC = 0.93 (95% CI: 0.69–0.98), for the object control score: ICC = 0.98 (95% CI: 0.93–0.99) and for total motor score (MS): ICC: 0.96; (95% CI: 0.82–0.99) were observed. The locomotion and object control scores were based on the presence (one) or absence (zero) of each of the performance criteria. For each subtest, the sum of the raw scores varied from 0–48 points.

### 2.3. Statistical Procedures

All variables were checked for normality using Kolmogorov–Smirnov tests. Descriptive analyses for continuous variables and frequency analyses for categorical variables were performed. A two-sided student’s *t-*test was used to compare mean values between genders and Cohen’s d was used to assess effect size [26]. chi-squared Test was used to calculate the association between proportion of guideline adherence and age. The level of significance was set at alpha level of 0.05. Data were analyzed using SPSS Windows v 20.0 (SPSS, Inc., Chicago, IL, USA).

A network analysis was used to assess the association between adherence to the 24-h movement guidelines and FMS, considering children’s age and BMI. The betweenness, closeness and strength centrality indicators were reported. Variables with higher betweenness values are more sensitive to changes and may act as a hub, connecting other pairs of variables in the network. A variable with a high closeness value will be quickly affected by changes in any part of the network and may also affect other parts. The strength indicator is essential to understand which variables present the most robust connections in the current network pattern.

The “Fruchterman–Reingold” algorithm was applied; therefore, data were shown in the relative space in which variables with stronger permanent statistics together and with less strongly applied variations repelled one another [27]. To improve the accuracy of the network we used the model “random fields of pair wise Markov”. The algorithm adds a “L1” (regularized neighborhood regression) penalty. The regulation is estimated by a less complete selection and contraction operator (Lasso) that controls the sparse network. The extended Bayesian information criterion (EBIC) to select the Lambda of the regularization parameter was observed. EBIC uses a hyperparameter (y) that determines how much EBIC selects sparse models [28,29]. We determine the y value at 0.25 (range from 0 to 0.50), which is a more parsimonious value when we have exploratory networks, as in the present study. The network analysis uses Least absolute shrinkage and selection operator (LASSO) regularized algorithms to obtain the precision matrix (weight matrix—see Supplementary Table S1). This matrix, when standardized, represents the associations between the variables present in the network. For a better visualization of the weight matrix, the network is presented in a graph that includes the variables (nodes) and the relations (lines). The blue color represents positive associations and the red color represents negative ones. The thickness and intensity of the colors represent the magnitude of the associations. The qgraph package of RStudio was used [30].

### 2.4. Ethical Aspects

All the Helsinki Declarations’ ethical aspects were followed [31]. The evaluation methods and procedures were approved by the Research Ethics Committee of Health Science Center of Federal University of Paraiba (protocol n. 2.727.698) and by the Education Board of João Pessoa city.

## 3. Results

In general, the participants physical characteristics (body weight, body height and BMI) were similar between sexes. Significant differences were seen for MVPA and FMS scores in 3-year-old children. The participants were engaged in almost 60 min/day of MVPA (57.93 ± 24.04) and were exposed to more than twice the recommended screen time (167.50 ± 99.07) (Table 1).

**Figure 1:**
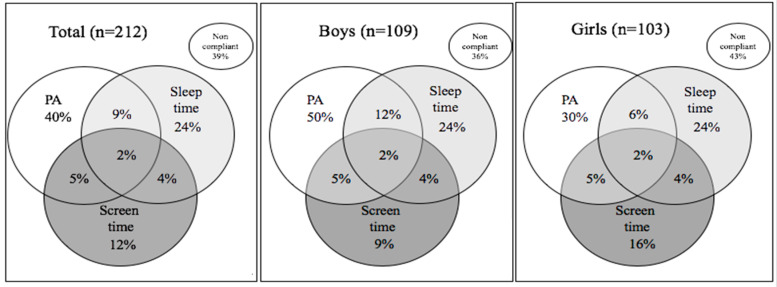
Venn diagrams showing the percentage of preschoolers compliant and not compliant with 24-h movement behaviors and the combinations of these guidelines for the overall sample and by sex.

Overall, regardless of sex, only 2% of the entire sample complied with the overall 24 h movement behaviors recommendations. PA was the behavior with higher compliance (40%). Data also showed that half of the boys are compliant with PA recommendations, although a lower percentage of them comply with the recommended amount of time sleep, when comparing to girls (Figure 1).

When analyzing preschoolers’ compliance with the guidelines by age, there was no association between proportion of children complying and their age group. The percentage of compliance with PA recommendations was higher for 5-year-old children, whose are also those that presented the lowest percentage of compliance with sleep and screen time (Table 2).

### 3.1. Network

**Figure 2:**
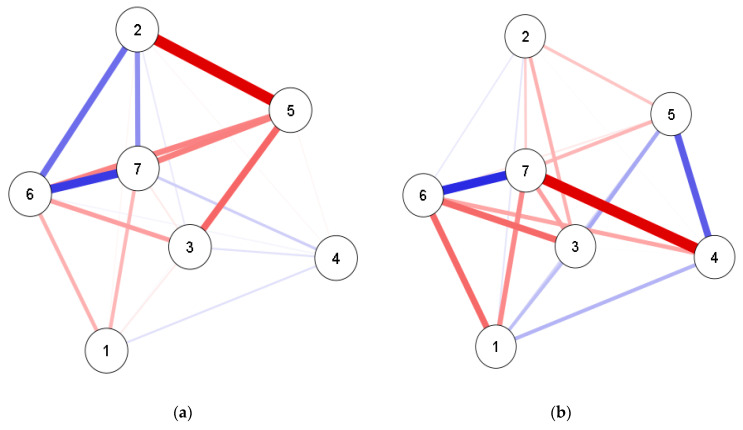

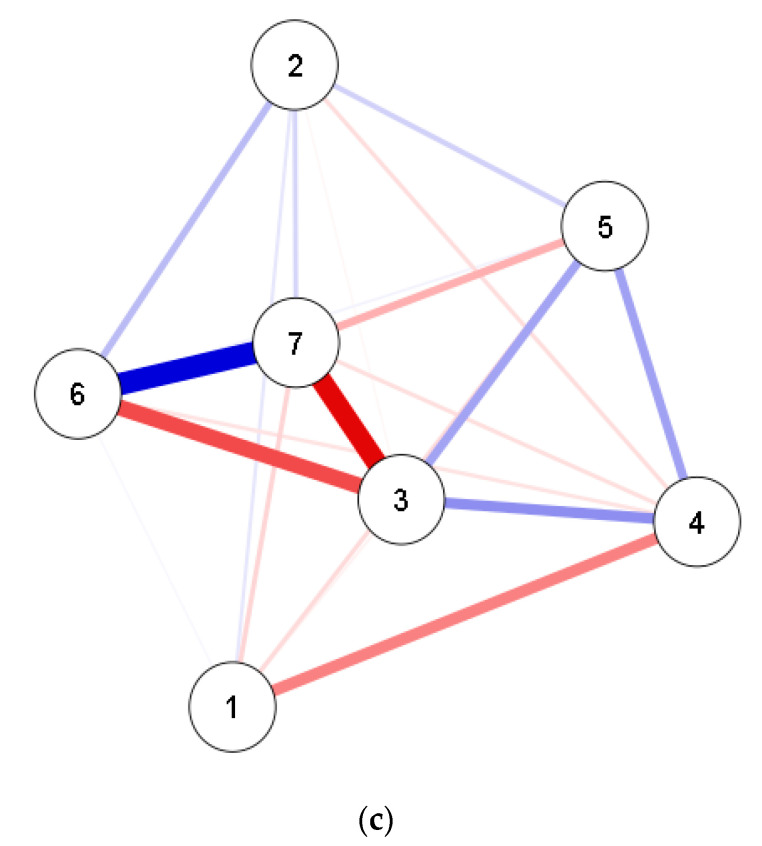
Network associations between movement behaviors, fundamental motor skills (FMS), Body Mass Index (BMI) and sex by age. (**a**) 3 year-olds; (**b**) 4 year-olds; (**c**) 5 year-olds; Positive associations are expressed by the blue color and negative associations by the red color. Thickness of the graph indicates the weight of the ratio. 1—BMI; 2—TPA + MVPA; 3—sleep time; 4—screen time; 5—sex; 6—locomotor skills; 7—ball skills.

The main results of the network analysis indicated that for the three age groups, the relationship between locomotion and ball skills scores was positive, with an association value greater than 0.50. In 3-year-old children, locomotion and ball skills showed positive relationships with PA adherence (TPA + MVPA), at 4 years-old, this relationship was weakened but remained significant, and at 5 years-old, it became negative with ball skills. The increase in sleep time adherence among the 3-year-old children was associated with the decline in the locomotion and ball skills scores. This relationship was more strongly significant for 4 and 5 year-olds. Regarding screen time, for 3 year-olds, the relationship with locomotion and object control was positive, but weak. For 4 and 5 year-olds, this relationship became negative and with more strength for 5 year-olds (Figure 2; Supplementary file 1).

### 3.2. Centrality Measures

The betweenness centrality indicator differed among ages. The locomotion (1.269) and ball skills (1.269) showed greater betweenness value to 3-year-old children. In 4-year-old children, the highest value was seen for adherence to screen time recommendations (1.569), and for those at 5 years-old, the highest value was seen for ball skills score (1.773). Regarding the closeness measure, locomotion (1.027) at 3 years-old, sleep time (1.278) at 4 years-old and ball skills (1.454) at 5 years-old presented the highest values. For strength, sex (1.044) was the variable most important in the network at 3 years-old, ball skills (1.307) and adherence to sleep recommendations (1.043) at 4 and ball skills (1.565) at 5 years-old (Table 3).

## 4. Discussion

Although prior studies have examined the association between daily movement behavior and FMS in preschoolers [14,32,33], the current study offers unique insight into these relationships, considering the compliance with the 2-h movement behaviors, using network analysis.

Our results showed that only two percent of the entire sample were compliant with the integrated movement behaviors guidelines. This prevalence is considerably lower than previously observed [4], and should be considered a matter of concern, given that more than a half of the entire sample were not compliant with PA recommendations, which is below the prevalence registered in other populations (i.e., 93.1% for Australians and 61.8% for Canadians). Although, compliance with PA recommendations can vary substantially across countries, the results of the current study are considerably lower than those reported previously. A study with young Belgian children showed that approximately 17.6% were compliant with PA recommendations [6] during weekdays and a recent study with more than seven hundred Portuguese preschoolers showed a prevalence of 28.6% compliance [7]. Methodological issues, such as different types of PA monitors, different PA cut-points or data validation criteria used may be significant contributors to the observed discrepancies [6]. Moreover, the low socioeconomic status (SES) of the Brazilian children, comparatively, may be a significant factor in such discrepancy. Interestingly, however, it has been shown that lifestyle factors can mediate the impact of SES on health indices [34], highlighting the need for careful consideration of numerous factors. Nevertheless, the relative impact of SES on movement guideline adherence is speculative and requires further investigation. Furthermore, even considering that the average sleep time (almost 9.5 h/day) observed is quite close to the recommended 10 h/day, less than a quarter (24%) of our sample were compliant with sleep time recommendations, which is extremely lower than the average percentage previously reported in other studies (94.3% for Belgians, 88.7% for Australians, 80.8% for Portuguese and 83.9% for Canadians) [4,5,6,7]. This finding may expose Brazilian to a higher screen time throughout the day in comparison to that which has been reported for other countries [4].

The low compliance observed for all the integrated guidelines in the current study may have a direct or indirect impact on their motor performance. Indeed, the current study showed that the association between adherence to the different movement behavior recommendations and FMS varies with developmental trajectory in early childhood. Our main findings indicated a positive association between adherence to PA recommendations and FMS scores in three and four-year-old children. At five years-old, this association is positive and weak for locomotion skills, but negative and weak for ball skills scores, which is concordant with previous observations During early childhood, FMS develop as a function of physical maturation, practice [35] and environmental opportunities [9]. Indeed, this is a sensitive period, when the impact of age on FMS is stronger [36] and potentialized as a consequence of a dynamic relationship with PA [10].

Nonetheless, these findings should be interpreted with caution, as children present greater PA [5,6,7,33] and FMS [35] variabilities, even at young ages. Although higher competence in FMS, such as run, throw, jump and catch, for example, has been highlighted as a precondition for functioning in daily life and participation in later physical or sport-specific activities [10], from a constrains-led perspective, skills acquisition is characterized as a dynamic movement system. Children need to develop a stable foundation of coordination to satisfy constrains in unpredictable contexts [37]. Learning when to coordinate their movements, children are able to explore, discover and stabilize movement function and, as a consequence [38], enhance their PA, suggesting that, as FMS proficiency increases, involvement in a greater and more diverse PA possibilities is likely.

As our data demonstrated, at five years-old, other social and environmental factors, such as adherence to screen time recommendations, may be more strongly and negatively associated with children’s FMS than adherence to PA recommendations. Indeed, the sedentary electronic lifestyle of young children, characterized by the excessive use of screen devices [39] may, at least partly, explain the negative association observed between FMS and screen time.

In addition, our data also showed that as children get older (five years-old), the strength of the negative association between sleep adherence and FMS ameliorates. Longer awake periods afford older children greater opportunities for movement experiences. It is well known that achieving an adequate amount of sleep plays a fundamental role in the memory consolidation process and in acquisition and retention of information [15]. Hence, as children grow and stabilize their sleep patterns, this negative association between sleep and FMS tends to be weaker. Moreover, from the network perspective, the relationship between pairs of variables, such as sleep and FMS, can be affected by all the other variables in the network. Thus, the negative association between BMI and FMS, for example, also affects the negative relationship between sleep time and FMS.”

Likewise, the emerged network reported in the present study emphasized ball skills as the most critical centrality variable, according to age (betweenness at three years-old; strength at four years-old; and betweenness, closeness and strength at five years-old). It is known that ball skills require children’s perceptual skills, such as perception of time and space, peripheral vision, anticipation and body kinesthetic perception and are important skills to break the barrier of proficiency postulated by Seelfeld [40]. Moreover, a recent systematic review study reported the existence of moderate relationships between ball skills and MVPA [13]. Thus, our results reinforce the importance of ball skills for the engagement and maintenance of children in physical and sports activities over time [41].

We also postulate, based on our findings, that the variation in screen time and sleep time can alter the relationship between ball skills and MVPA at different ages. It seems somehow logical that movement behaviors, FMS and BMI are closely related. Indeed, FMS may be a consequence of PA levels [42] and movement behaviors [41] may be determinants for a healthy BMI.

Moreover, considering children’s low variance in BMI, this variable did not show strong associations with FMS in three and four-year-old children. This does not mean BMI is not important, but that in our specific context, BMI did not yield sufficient influence in the emerged network. Nonetheless, for five-year-old children, data showed a stronger negative association between BMI and FMS. The mediating participation of body weight status in the relationship between PA and FMS has been theoretically reported [10], although the existing literature remains equivocal [43,44]. Actually, some studies have reported an association between mastery in FMS and obesity rates [45,46]. Thus, our findings further highlight that the negative association between BMI and FMS should be a matter of concern even at young ages.

The main strength of our study is the utilization of a novel network perspective to understanding the association of adherence to different movement behaviors and FMS in preschoolers. This perspective permits evaluation of the interactions between variables as a complex system, considering its nonlinearity and the single role of each variable in the network, based on centrality measures [47]. We can also maintain in the analysis variables that present small effects, since in complex systems, a small effect can be responsible for important changes in the entire network [17]. Moreover, this perspective is in line with recent conceptual models when suggesting that PA and FMS are complex variables, as they are affected by factors of various natures, at different levels, in different areas [48]. In this sense, this study demonstrates a new statistical and theoretical perspective to understand the associations of movement behavior, FMS and correlates, respecting the innate complexity of these relationships.

Despite representing a novel addition to the literature, our study does have limitations that should be considered. Indeed, inherent to all studies of a cross-sectional nature, we are unable to make any causal inference from our data. In addition, the self-reported parental recall used in this study to assess sleep and screen time may be associated with over or underestimation, due its subjective nature. However, in practicality, ensuring preschool aged children comply with sensor wear at night is fraught with difficulty and represents a very real, ecological, barrier to objectively assessing sleep time. In addition, we did utilize objective measurement of movement behaviors during the day. Clearly, further work that considers the developmental and longitudinal aspects of PA, FMS and BMI should be conducted and subsequently analyzed using a network approach, which we have demonstrated.

## 5. Conclusions

The present study presents a novel statistical and theoretical perspective that permits insight into the associations between movement behavior, FMS and their correlates, that appropriately consider the inherent, multifaceted, complexity of these relationships. Descriptively, only two percent of the entire sample complied with the overall 24-h movement behaviors recommendations; while the emerged network in the present study emphasized ball skills as the most critical centrality variable, according to age, reinforcing the importance of ball skills for the engagement and maintenance of PA and sports in children.

## Figures and Tables

**Table 1:** Sample characteristics and differences by age and sex.

	3 Years Old		Value	4 Years Old		Value	5 Years Old		Value	Total
Categories	Boys (*n* = 39)	Girls (*n* = 35)	P d	Boys (*n*= 33)	Girls (*n* = 40)	P d	Boys (*n* = 37)	Girls (*n* = 28)	P d	*n* = 212
Age (years)	3.58 ± 0.30	3.67 ± 0.40	0.32 0.23	4.49 ± 0.28	4.45 ± 0.27	0.49 0.16	5.33 ± 0.27	5.44 ± 0.28	0.13 0.37	4.45 ± 0.77
Body weight (kg)	16.15 ± 1.86	16.33 ± 2.72	0.73 0.07	18.48 ± 3.05	17.36 ± 3.01	0.12 0.37	19.83 ± 2.96	20.30 ± 2.77	0.52 0.16	17.95 ± 3.14
Body height (cm)	100.25 ± 4.88	100.45 ± 6.18	0.87 0.03	107.07 ± 4.24	104.67 ± 4.90	0.032 0.52	112.44 ± 4.93	112.56 ± 4.20	0.91 0.02	105.90 ± 6.98
BMI	16.09 ± 1.30	16.14 ± 1.69	0.89 0.03	16.07 ± 1.87	15.80 ± 1.76	0.52 0.15	15.65 ± 1.86	16.00 ± 1.54	0.41 0.20	15.95 ± 1.67
TPA (min/day)	267.97 ± 61.21	252.53 ± 57.27	0.26 0.26	259.76 ± 75.20	286.76 ± 53.87	0.079 0.41	271.37 ± 68.19	277.86 ± 68.96	0.70 0.09	269.59 ± 64.25
LPA (min/day)	206.81 ± 51.12	208.78 ± 49.84	0.86 0.03	202.38 ± 62.92	227.02 ± 43.92	0.053 0.46	206.11 ± 48.51	218.36 ± 53.17	0.33 0.24	211.66 ± 51.64
MVPA (min/day)	61.16 ± 20.45	43.75 ± 19.68	<0.001 0.86	57.38 ± 19.79	59.74 ± 20.41	0.62 0.11	65.26 ± 32.34	59.50 ± 24.95	0.43 0.19	57.93 ± 24.04
Screen time (min/day)	173.94 ± 110.98	170.26 ± 126.54	0.89 0.03	172.34 ± 94.77	146.62 ± 83.19	0.22 0.29	189.67 ± 93.43	149.88 ± 72.02	0.06 0.46	167.50 ± 99.07
Sleep time (min/day)	581.53 ± 76.24	550.33 ± 68.67	0.07 0.42	560.86 ± 68.20	560.47 ± 78.03	0.98 0.00	563.95 ± 63.14	595.40 ± 71.35	0.06 0.47	567.95 ± 71.95
Locomotor	19.56 ± 8.80	14.51 ± 7.26	0.009 0.62	20.61 ± 6.18	21.00 ± 8.32	0.82 0.05	25.24 ± 7.98	24.36 ± 8.00	0.65 0.11	20.79 ± 8.49
Ball skills	17.28 ± 6.44	13.89 ± 4.86	0.013 0.59	18.27 ± 6.66	15.95 ± 7.23	0.16 0.33	21.03 ± 6.45	18.68 ± 7.05	0.16 0.35	17.46 ± 6.80
MC	36.85 ± 13.37	28.40 ± 10.68	0.004 0.69	38.88 ± 11.67	36.95 ± 14.21	0.53 0.14	46.27 ± 12.87	43.04 ± 13.57	0.33 0.24	38.25 ± 13.84

TPA—total physical activity; LPA—light physical activity; MVPA—moderate to vigorous physical activity; MC—motor competence; d—Cohen’s d.

**Table 2:** Percentage of guideline compliance among preschoolers by age.

Movement Behaviors	Adherence to 24-h Movement Behaviors	3 Years Old *n* (%)	4 Years Old *n* (%)	5 Years Old *n* (%)
TPA (≥ 180 min/day+ MVPA ≥ 60 min/day)	Compliant (%)	28 (37.8)	25 (34.2)	32 (49.2)
	Non-compliant (%)	46 (62.2)	48 (65.8)	33 (50.8)
screen time (≤60 min/day)	Compliant (%)	11 (14.9)	7 (9.6)	8 (12.3)
	Non-compliant (%)	63 (85.1)	66 (90.4)	57 (87.7)
sleep time (600–780 min/day)	Compliant (%)	17 (23.0)	18 (24.7)	16 (24.6)
	Non-compliant (%)	57 (77.0)	55 (75.3)	49 (75.4)

TPA—total physical activity; MVPA—moderate-to-vigorous physical activity.

**Table 3:** Centrality measures.

Age	Betweenness			Closeness			Strength		
	3	4	5	3	4	5	3	4	5
BMI	−0.781	−0.833	−1.048	−0.924	−1.190	−0.740	−1.072	−1.080	−0.269
TPA + MVPA	−0.781	−0.833	−1.048	0.430	−1.462	−1.653	0.460	−1.229	−1.632
Sleep time	−0.781	1.110	0.081	−0.348	1.278	0.338	−0.501	1.043	−0.268
Screen time	−0.781	1.596	0.645	−1.642	0.214	0.242	−1.447	−0.111	0.366
Sex	0.586	−0.833	−0.483	0.605	−0.069	−0.250	1.044	−0.472	−0.423
Locomotor	1.269	0.139	0.081	1.027	0.541	0.609	0.924	0.541	0.662
Ball skills	1.269	−0.347	1.773	0.852	0.687	1.454	0.593	1.307	1.563

BMI—body mass index; TPA—total physical activity; MVPA—moderate-to-vigorous physical activity.

## Supplementary Materials

The following are available online at https://www.mdpi.com/1660-4601/17/15/5443/s1, Table S1: Weights matrix.
