# Supplementary material for: Association between Compliance with the 24-Hour Movement Guidelines and Fundamental Movement Skills in Preschoolers: A Network Perspective
Source: Int J Environ Res Public Health. 2020 Jul 28;17(15):5443. doi: 10.3390/ijerph17155443 (PMC7652226; doi:10.3390/ijerph17155443)
Supplement: Supplementary file 1 [file ijerph-17-05443-s001.pdf]

Supplementary file

Table S1. Weigths Matrix.

	3 years-old							4 years-old							5 years-old						
**Variables**	**1**	**2**	**3**	**4**	**5**	**6**	**7**	**1**	**2**	**3**	**4**	**5**	**6**	**7**	**1**	**2**	**3**	**4**	**5**	**6**	**7**
1	0,00							0,00							0,00						
2	-0,03	0,00						0,07	0,00						0,08	0,00					
3	-0,06	0,07	0,00					-0,03	-0,02	0,00					0,19	-0,23	0,00				
4	0,10	-0,03	0,09	0,00				-0,32	-0,09	0,29	0,00				0,23	0,01	0,01	0,00			
5	0,02	-0,75	-0,45	-0,03	0,00			-0,10	0,12	0,24	0,24	0,00			0,13	-0,17	0,26	0,47	0,00		
6	-0,24	0,43	-0,29	0,04	-0,37	0,00		0,02	0,18	-0,46	-0,08	0,03	0,00		-0,43	0,07	-0,43	-0,27	-0,07	0,00	
7	-0,22	0,33	-0,07	0,15	-0,38	0,57	0,00	-0,11	0,11	-0,61	-0,09	-0,21	0,65	0,00	-0,34	-0,15	-0,28	-0,74	-0,22	0,60	0,00

1= BMI; 2 = TPA+MVPA; 3 = Sleep time; 4 Screen time; 5 = Sex; 6 = Locomotor Skills; 7 = Object control Skills.
